# Supplementary material for: Heavy water inhibits DNA double-strand break repairs and disturbs cellular transcription, presumably via quantum-level mechanisms of kinetic isotope effects on hydrolytic enzyme reactions
Source: PLoS One. 2024 Oct 3;19(10):e0309689. doi: 10.1371/journal.pone.0309689 (PMC11449287; doi:10.1371/journal.pone.0309689)
Supplement: S1 Text — (PDF) [file pone.0309689.s001.pdf]

Supporting Information Text S1

According to the textbook by P.W. Atkins [25,26], the quantum tunneling probability of the transmitted wave in S1B Fig is calculated by using the Schrödinger equation, as follows.

The Schrödinger equation is:

$$-(\hbar^2/2m)\, d^2\phi/dx^2 + V\phi = E\phi$$
$$\hbar = h/2\pi$$

where ϕ (wave function), E (total energy: motion + potential), *m* (mass of the particle) *h* (Planck's constant), and V (potential energy of the particle).

In fig. S1B, the Schrödinger equations for each region are:
Region A (V = 0): $-(\hbar^2/2m)\, d^2\phi/dx^2 = E\phi$
Region B (V > 0): $-(\hbar^2/2m)\, d^2\phi/dx^2 + V\phi = E\phi$
Region C (V = 0): $-(\hbar^2/2m)\, d^2\phi/dx^2 = E\phi$.

The solutions of these equations are:
Region A: $\phi_A(x) = Ae^{ikx} + A'e^{-ikx}$ $\quad k = (2mE/\hbar^2)^{1/2}$
Region B: $\phi_B(x) = Be^{k\prime x} + B'e^{-k\prime x}$ $\quad k' = (2m(V-E)/\hbar^2)^{1/2}$
Region C: $\phi_c(x) = Ce^{ikx} + C'e^{-ikx}$ $\quad k = (2mE/\hbar^2)^{1/2}$.

Since there are no particles moving in the − x direction on the right side, $C' = 0$.
The probability of the quantum tunneling effect, *P*, where a particle passes through a barrier, is

$$P = |C|^2/|A|^2$$

Since ϕ and its derivative $d\phi/dx$ are continuous on the left ($x = 0$) and right (x = L) sides,
$(\phi_A(0) = \phi_B(0);\ (\phi_A/dx)_{x=0} = (\phi_B/dx)_{x=0}$
$\phi_B(L) = \phi_C(L);\ (\phi_B/dx)_{x=L} = (\phi_C/dx)_{x=L}$.
Therefore,
$A + A' = B + B';\ ikA - ikA' = Bk' - B'k'$
$Be^{k\prime L} + B'e^{-k\prime L} = Ce^{ikL};\ k'Be^{k'L} - k'B'e^{-k\prime L} = ikCe^{ikL}$

$$P = 1/(1+G)$$

$$G = \frac{[\exp[\{2m(V-E)/\hbar^2\}^{1/2}L] - \exp[-\{2m(V-E)/\hbar^2\}^{1/2}L]]^2}{4\,(E/V)\{1-(E/V)\}}$$

If E < V; e.g., E/V = 0.5, then $4\,(E/V)\{1-(E/V)\} = 1$.
Comparing ^{1}H and ^{2}H, we get

$$\exp[\{2\times 2\times (V-E)/\hbar^2\}^{1/2}L] > \exp[\{2\times 1\times (V-E)/\hbar^2\}^{1/2}L]$$

$$\exp[-\{2\times 2\times (V-E)/\hbar^2\}^{1/2}L] < \exp[-\{2\times 1\times (V-E)/\hbar^2\}^{1/2}L].$$

Therefore,

$$G_{^1H} < G_{^2H}$$

and

$$P_{^1H} > P_{^2H}$$

Thus, ^{1}H has a higher probability of quantum tunneling than ^{2}H.

These formulas clarify that hydrogen has a higher probability of quantum tunneling than deuterium, and that the kinetic isotope effect occurs via the different probabilities of quantum tunneling between hydrogen and deuterium. In addition, as described in Atkins' Physical Chemistry, the probability of quantum tunneling (*P*) decreases exponentially with $m^{1/2}$. Therefore, quantum tunneling is more difficult for heavier particles [25,26]. At higher reaction temperatures, E > V, the differences in quantum tunneling effects due to differences in particle mass disappear. Therefore, the kinetic isotope effect due to quantum tunneling is temperature dependent.
