# Supplementary material for: Heavy water inhibits DNA double-strand break repairs and disturbs cellular transcription, presumably via quantum-level mechanisms of kinetic isotope effects on hydrolytic enzyme reactions
Source: PLoS One. 2024 Oct 3;19(10):e0309689. doi: 10.1371/journal.pone.0309689 (PMC11449287; doi:10.1371/journal.pone.0309689)
Supplement: S2 Text — (PDF) [file pone.0309689.s002.pdf]

Supporting Information Text S2

As described in Atkins' Physical Chemistry [25,26], the kinetic isotope effect is also caused by differences in the zero-point vibrational potential energies of the chemical bonds involved in the reaction, which are likewise related to quantum theory (S1C Fig).

The vibrational potential energy of a chemical bond involved in a reaction is described as follows.

$$\nu = \frac{1}{2\pi}\sqrt{\frac{\kappa}{\mu}}$$

(where ν is the vibration frequency of the chemical bond between two atoms, and κ is the force constant of the chemical bond),

$\mu = (m \times M) \div (m + M)$

(where μ is the reduced mass, and m and M are the masses of the two atoms), and

$$E_n = \left(n + \frac{1}{2}\right) h\nu$$

(where E_n is vibrational potential energies according to the quantum mechanics, n = 0, 1, 2, 3, etc., and *h* is the Planck constant).

Therefore, the lowest vibrational potential energy is described as below.

$$E_0 = \frac{1}{2} h\nu = \frac{h}{4\pi}\sqrt{\frac{\kappa}{\mu}}$$

(where E_0 is the vibrational zero-point energy).

Accordingly, the zero-point energy (E_0) is decreased when the reduced mass (μ) is increased, and the reduced mass is increased when the mass of one of the atoms involved in the chemical reaction is increased. Assuming that one of the atoms is hydrogen (H), the E_0 value is changed at both the GS and TS when the hydrogen is substituted with its heavier isotope deuterium (D) (Figure 1c). Therefore, the activation energy (ΔE) is increased by the substitution of H with D, thereby decreasing the reaction rate by the substitution.

The ratio of the reaction rate constants between the cleavage of O-H and O-D bonds, *k*(O-D)/*k*(O-H), is shown by the following formulas [26].

$$\frac{k\,(O-D)}{k\,(O-H)} = e^{-\lambda}$$

with

$$\lambda = \frac{hc\nu(O-H)}{2kT}\left\{1 - \left(\frac{\mu_{OH}}{\mu_{OD}}\right)^{1/2}\right\}$$

Where h is Planck's constant, c is the speed of light, ν(O-H) is the vibrational wavenumber of the O-H bond, μ_{OH} and μ_{OD} are the relevant effective masses, k is the Boltzmann constant, and T is the absolute temperature. Therefore, the kinetic isotope effect is caused by the differences in the relevant effective masses between OH and OD. In addition, the formulas indicate that k(O-D)/k(O-H) depends on temperature. However, comparing k(O-D)/k(O-H) at 15°C (288K) and 42°C (315K), $e^{-\lambda}$ (at 315 K)/$e^{-\lambda}$ (at 288 K) = 1.000297663340558. Thus, in the temperature range used in our experiments, the isotope effects caused by the differences in zero-point vibrational potential energy are less sensitive to temperature variation.
