# Supplementary material for: Heavy water inhibits DNA double-strand break repairs and disturbs cellular transcription, presumably via quantum-level mechanisms of kinetic isotope effects on hydrolytic enzyme reactions
Source: PLoS One. 2024 Oct 3;19(10):e0309689. doi: 10.1371/journal.pone.0309689 (PMC11449287; doi:10.1371/journal.pone.0309689)
Supplement: S1 Fig — (A) Conceptual image of quantum tunneling, in which the chemical reaction of the reactant (brown circle) at the ground state can proceed without reaching the transition state by penetrating through the energy barrier. (B) Conceptual diagram of quantum tunneling with a 1D box barrier model (1). The height of the potential barrier (V), the thickness of the potential barrier (L), and the incident wave, reflected wave, and transmitted wave are shown. (C) Difference in zero-point vibrational energies between hydrogen (H) and deuterium (D) to explain the kinetic isotope effect (2–4). (D) Chemical reactions of SIRT3-mediated deacetylation of an acetylated lysine residue in the presence of H2O or D2O. (PDF) [file pone.0309689.s003.pdf]

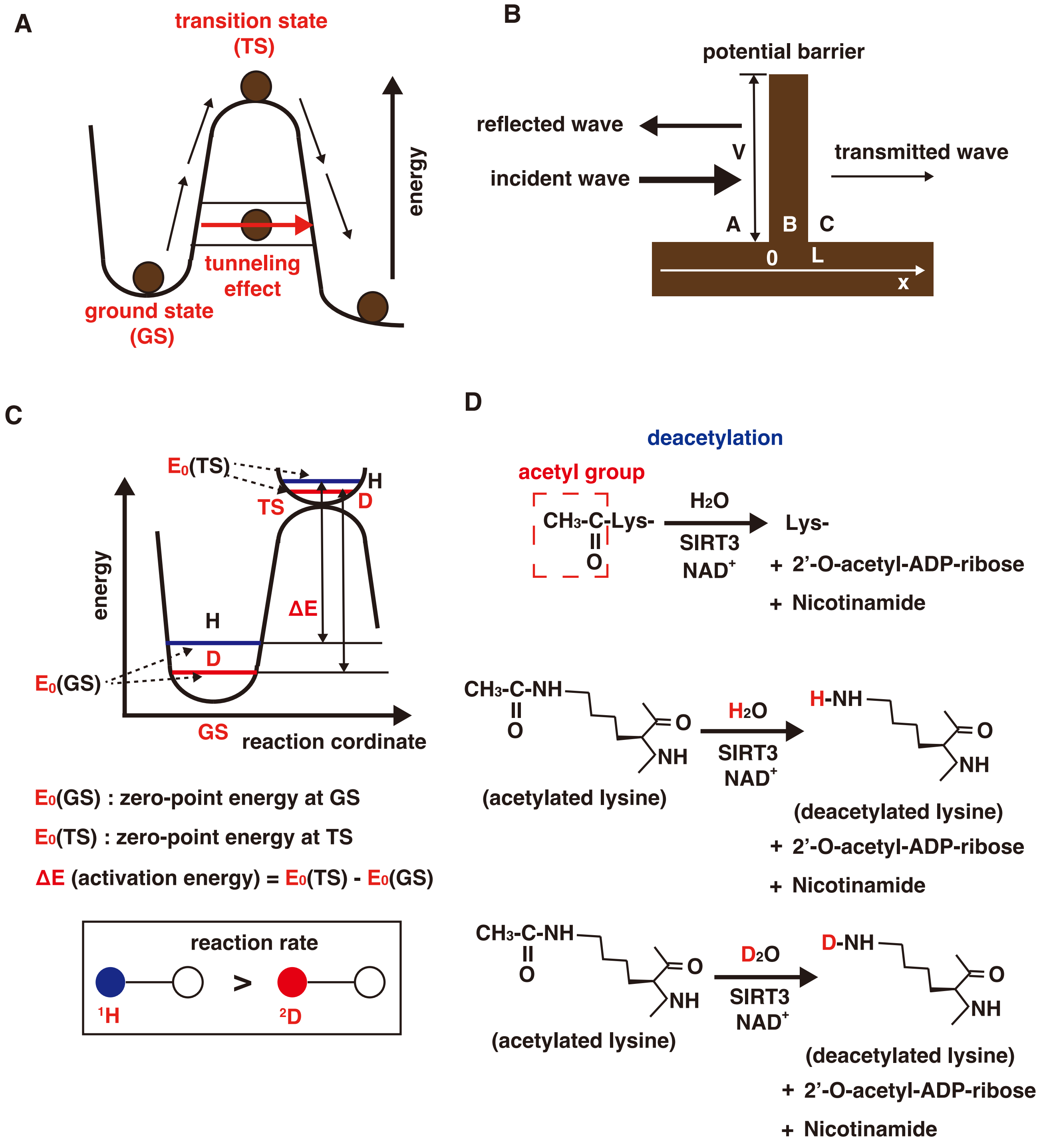
A
transition state
(TS)
energy
tunneling
effect
ground state
(GS)
B
potential barrier
reflected wave
incident wave
transmitted wave
V
A
B
C
0
L
x
C
E0(TS)
H
D
TS
energy
ΔE
H
D
E0(GS)
GS
reaction cordinate
E0(GS) : zero-point energy at GS
E0(TS) : zero-point energy at TS
ΔE (activation energy) = E0(TS) - E0(GS)
reaction rate
>
1H
2D
D
deacetylation
acetyl group
CH3-C-Lys-
O
H2O
SIRT3
NAD+
Lys-
+ 2'-O-acetyl-ADP-ribose
+ Nicotinamide
CH3-C-NH
O
O
NH
(acetylated lysine)
H2O
SIRT3
NAD+
H-NH
O
NH
(deacetylated lysine)
+ 2'-O-acetyl-ADP-ribose
+ Nicotinamide
CH3-C-NH
O
O
NH
(acetylated lysine)
D2O
SIRT3
NAD+
D-NH
O
NH
(deacetylated lysine)
+ 2'-O-acetyl-ADP-ribose
+ Nicotinamide

Fig. S1.
