# Supplementary figures and images for: Heavy water inhibits DNA double-strand break repairs and disturbs cellular transcription, presumably via quantum-level mechanisms of kinetic isotope effects on hydrolytic enzyme reactions
Source: PLoS One. 2024 Oct 3;19(10):e0309689. doi: 10.1371/journal.pone.0309689 (PMC11449287; doi:10.1371/journal.pone.0309689)

**A**

*In vitro* HDAC assay

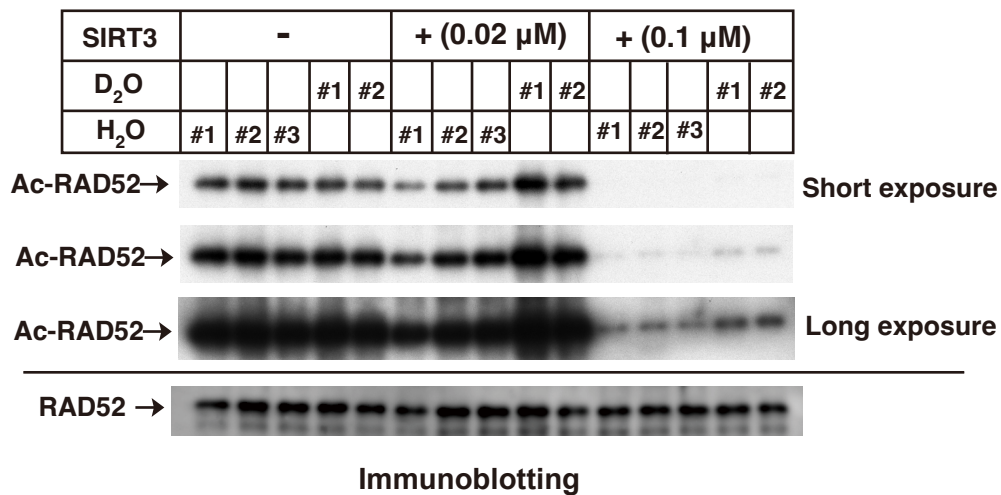

**B**

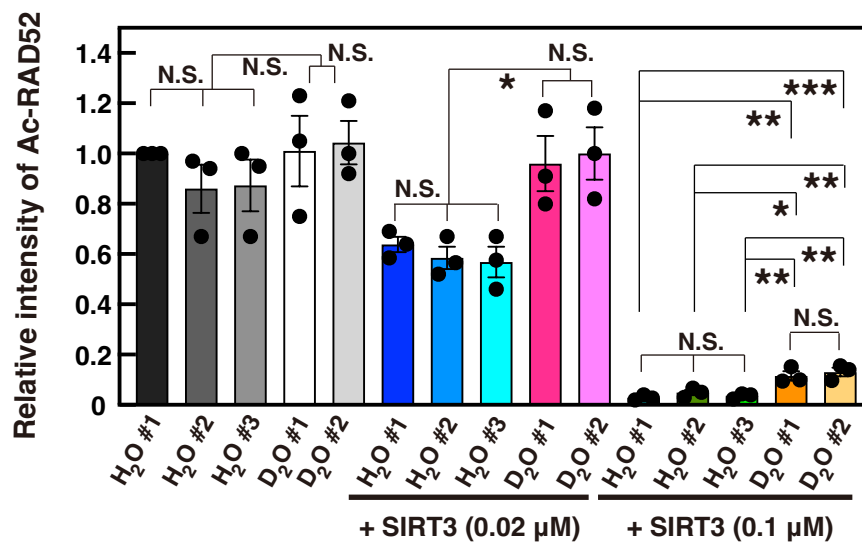

**Fig. S2.**

Supplement: S2 Fig — (A) In vitro acetylation assays were performed as described in Fig 1, in the presence of H2O or D2O purchased from different sources. H2O #1, H2O #2, H2O #3, D2O #1, and D2O #2 were used, as described in the Materials and Methods. After the addition of a poly dT 68 mer, an aliquot of the reaction mixture containing the RAD52 protein (final concentration 0.08 μM) was incubated with the indicated amount of SIRT3 in HDAC buffer, prepared with H2O or D2O from different manufacturers, at 30˚C for 60 min. The reaction mixtures were subjected to SDS-PAGE, followed by immunoblotting with an anti-acetylated lysine antibody (Ac-RAD52) and an anti-RAD52 antibody (bottom, RAD52). The long and short exposures are also presented for the immunoblotting with an anti-acetylated lysine antibody. (B) The relative band intensities of acetylated RAD52 normalized to those of the RAD52 bands are shown in the graph. The mean values and standard errors of the mean from 3 independent experiments were plotted, with dots of each data values. For each SIRT3 concentration, the samples connected by lines were compared (*P <0.05, **P <0.01, **P <0.001 and N.S., not significant by one-way ANOVA with Dunnet’s post hoc test with the KaleidaGraph software). (PDF) [file pone.0309689.s004.pdf]

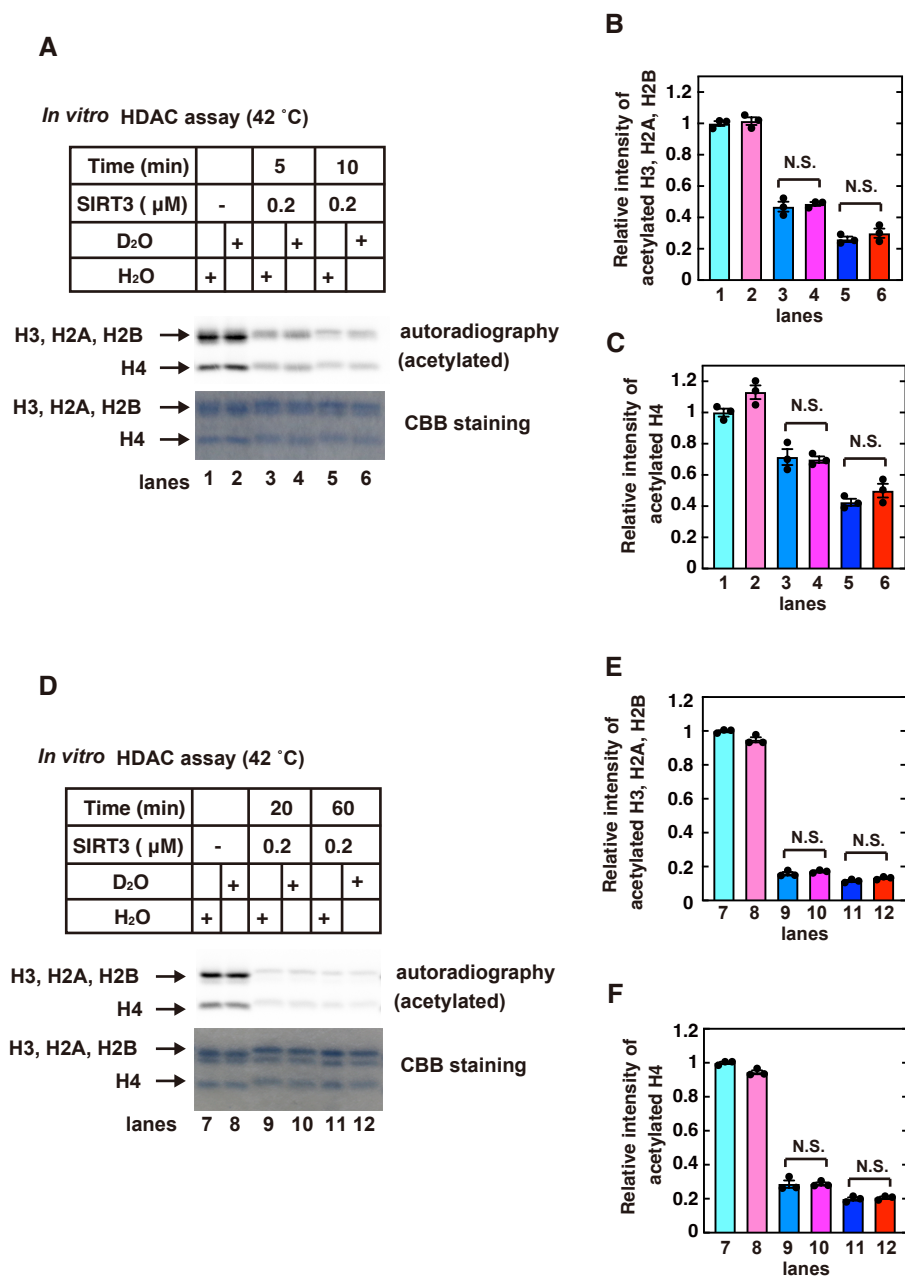

**Fig. S3.**

Supplement: S3 Fig — (A and D) In vitro acetylation and deacetylation assays of histone proteins were performed, as described in the Materials and Methods. The deacetylation reactions were performed with the indicated amount of SIRT3 at 42˚C for the indicated times. The reaction mixtures were subjected to SDS-PAGE, followed by CBB staining (bottom) and autoradiography (top, acetylated proteins). (B, C, E, and F) The relative band intensities of acetylated H3, H2A, and H2B (B and E) and acetylated H4 (C and F) are shown in the graph. Mean values and standard errors of the mean from 3 independent experiments were plotted, with dots of each data values. The samples connected by lines were compared (N.S., not significant by an unpaired Student´s t-test). (PDF) [file pone.0309689.s005.pdf]

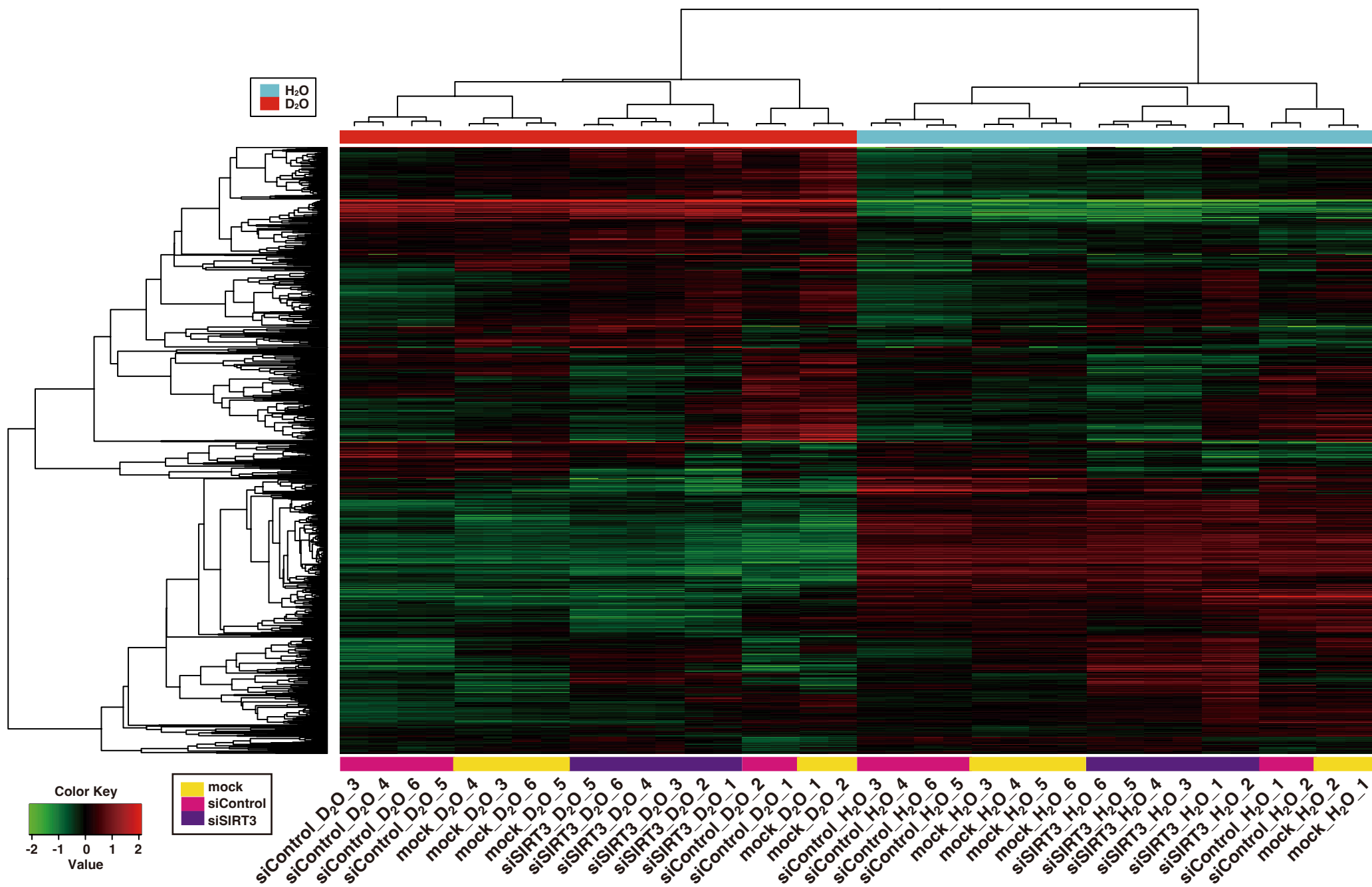

**Fig. S4.**

Supplement: S4 Fig — Hela pDR-GFP cells untreated (mock) or transfected with siRNA (siControl or siSIRT3) were cultured in medium made with H2O or D2O for 5h, and their RNA samples were subjected to an RNA-seq analysis. Six samples were used for each experimental condition. The heatmap was generated with iDEP96, as described in the Materials and Methods. (PDF) [file pone.0309689.s006.pdf]

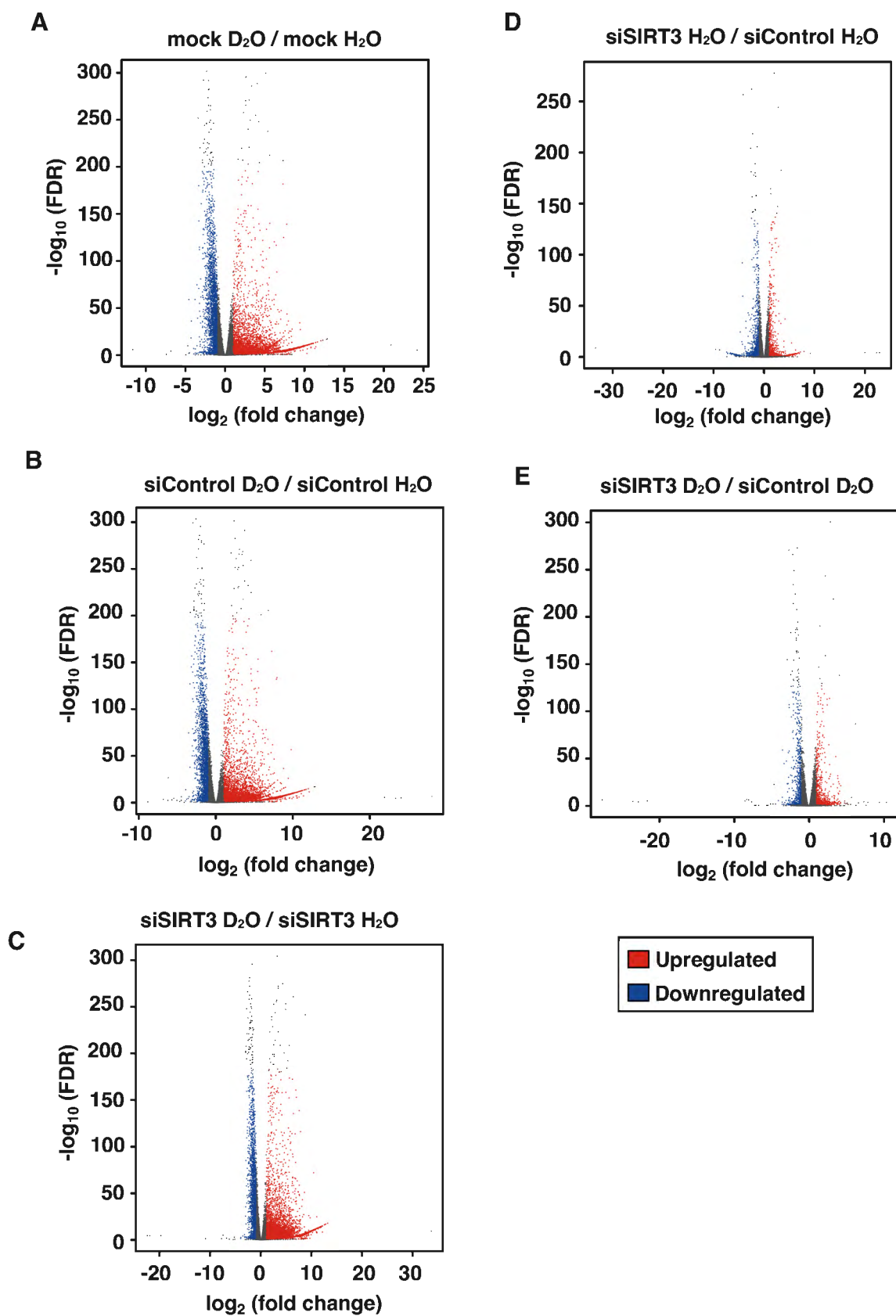

Fig. S5.

Supplement: S5 Fig — The RNA-seq data shown in S4 Fig were used for a volcano plot analysis with the DEG2 function of iDEP96. The log2 (fold change) versus -log10 (false discovery rate (FDR)) is shown in each plot. (PDF) [file pone.0309689.s007.pdf]

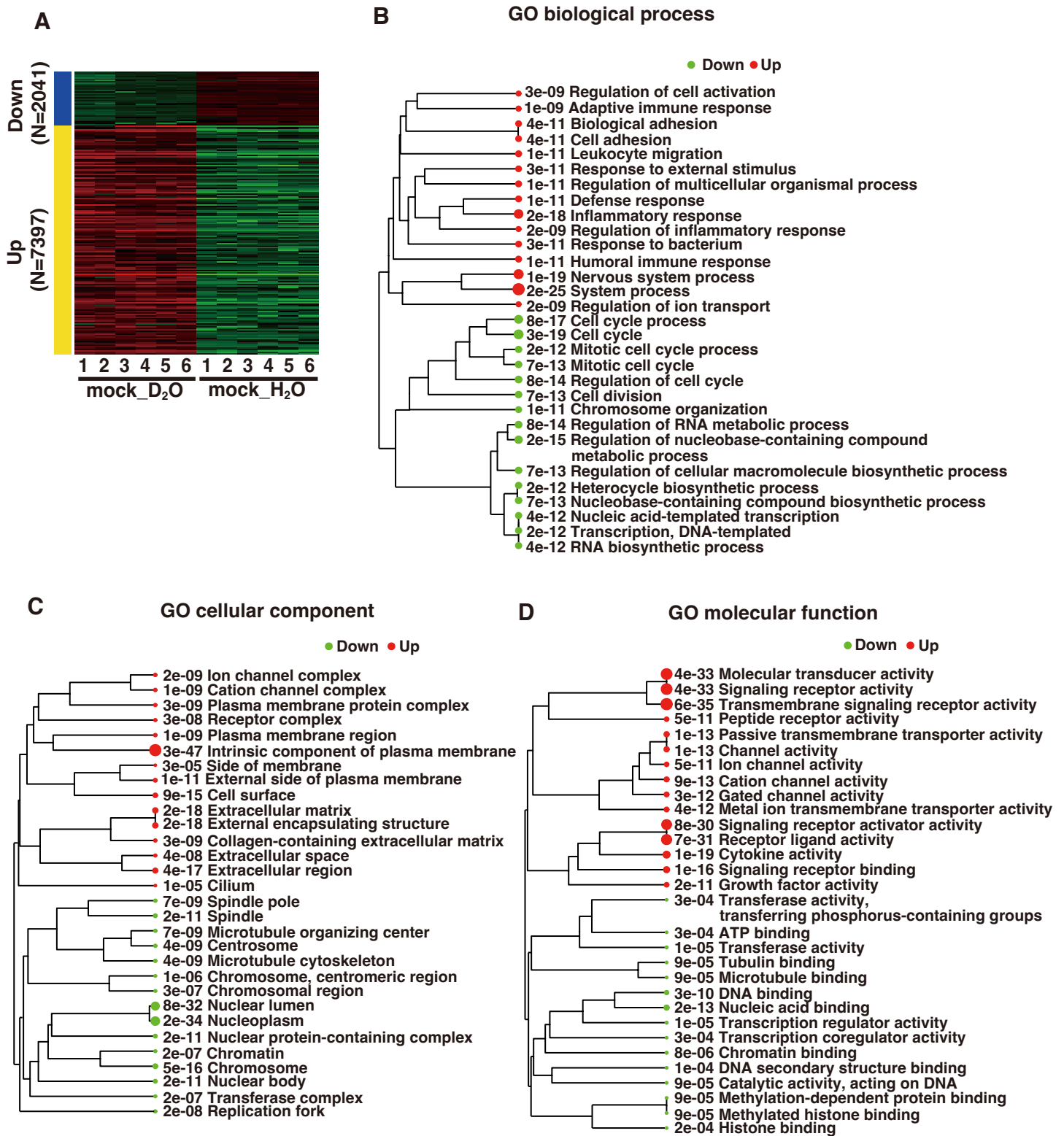

**Fig. S6.**

Supplement: S6 Fig — (A to D) The RNA-seq data shown in S4 Fig were used. The RNA-seq data were analyzed with the DEG2 function of iDEP96. Upregulated and downregulated genes are colored red and green, respectively. (A) Heatmap analysis of gene expression differences. (B to D) Enrichment trees. Enrichment pathway analyses were performed for three categories: GO biological process (B), GO cellular component (C), and GO molecular function (D). The false discovery rate (FDR) is shown and is also represented by the size of the circle. (PDF) [file pone.0309689.s008.pdf]

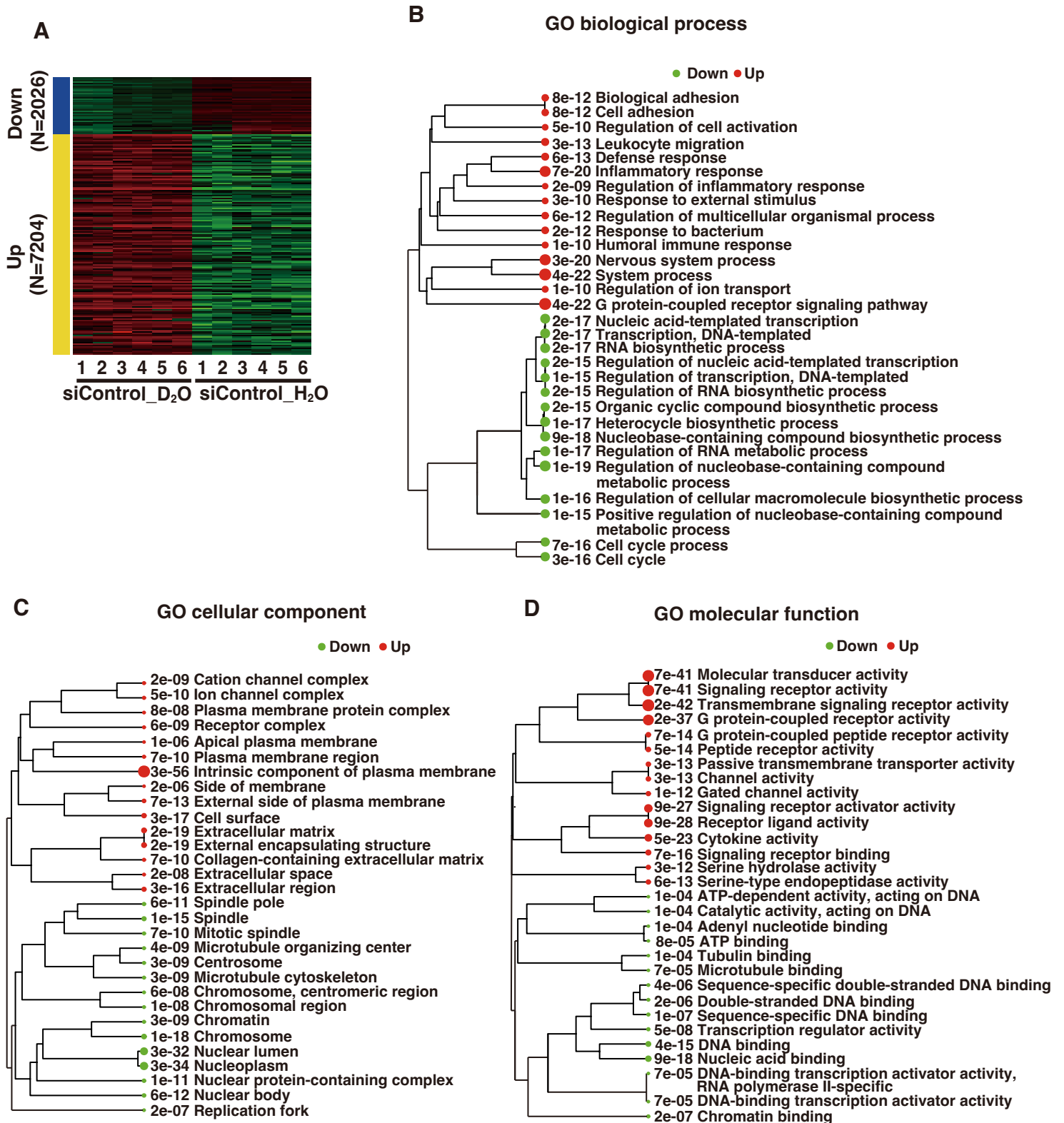

Fig. S7.

Supplement: S7 Fig — (A to D) The RNA-seq data shown in S4 Fig were used. The RNA-seq data were analyzed with the DEG2 function of iDEP96. Upregulated and downregulated genes are colored red and green, respectively. (A) Heatmap analysis of gene expression differences. (B to D) Enrichment trees. Enrichment pathway analyses were performed for three categories: GO biological process (B), GO cellular component (C), and GO molecular function (D). The false discovery rate (FDR) is shown and is also represented by the size of the circle. (PDF) [file pone.0309689.s009.pdf]

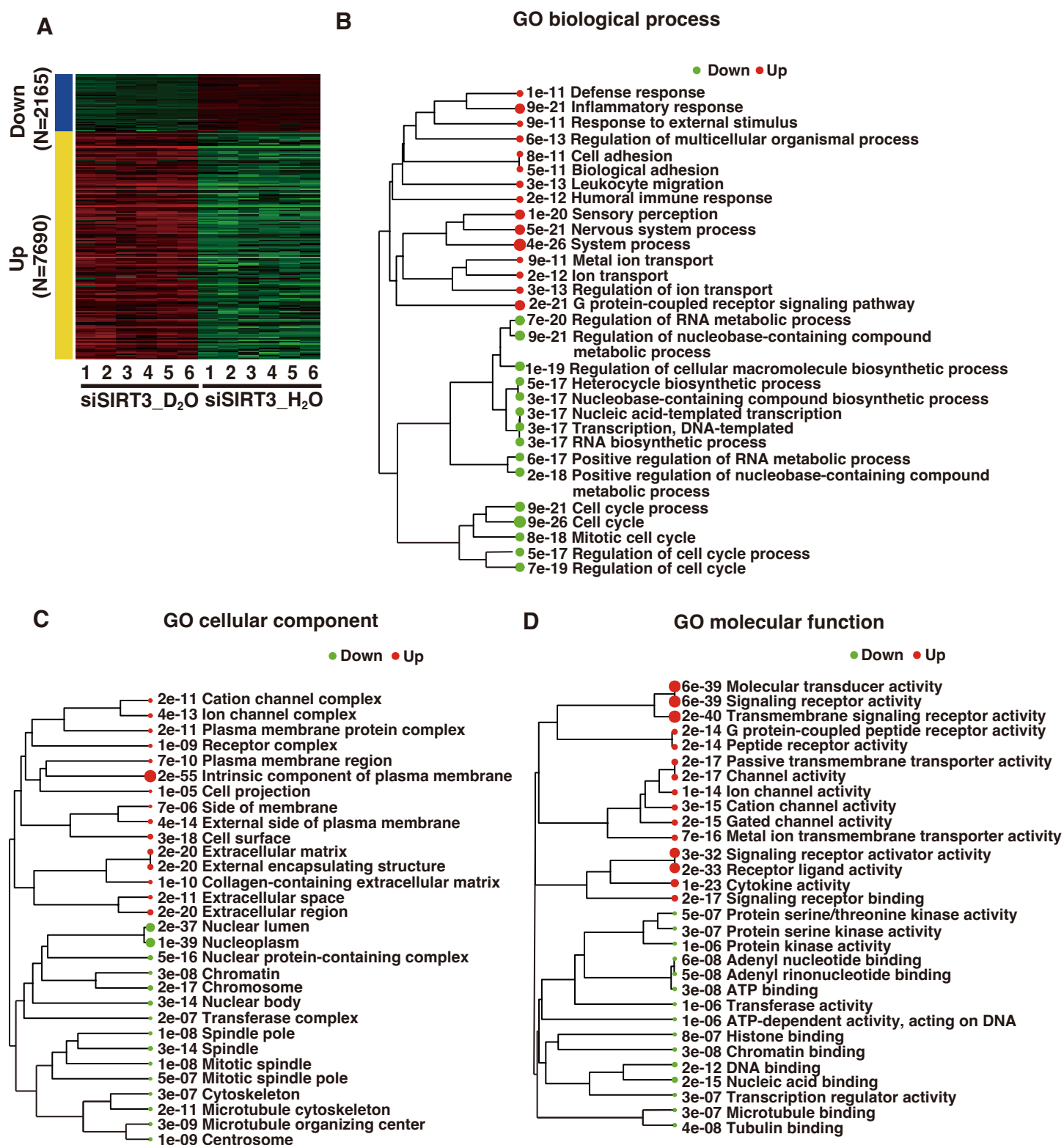

Fig. S8.

Supplement: S8 Fig — (A to D) The RNA-seq data shown in S4 Fig were used. The RNA-seq data were analyzed with the DEG2 function of iDEP96. Upregulated and downregulated genes are colored red and green, respectively. (A) Heatmap analysis of gene expression differences. (B to D) Enrichment trees. Enrichment pathway analyses were performed for three categories: GO biological process (B), GO cellular component (C), and GO molecular function (D). The false discovery rate (FDR) is shown and is also represented by the size of the circle. (PDF) [file pone.0309689.s010.pdf]

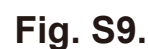

Supplement: S9 Fig — The RNA-seq data shown in S4 Fig were used. Expression levels of each gene were visualized on KEGG pathway map of “cytokine-cytokine receptor interaction”, as described in the Materials and Methods. The red and green colors, according to shading, show an increase and decrease in gene expression, respectively, with D2O treatment compared to H2O treatment. (PDF) [file pone.0309689.s011.pdf]

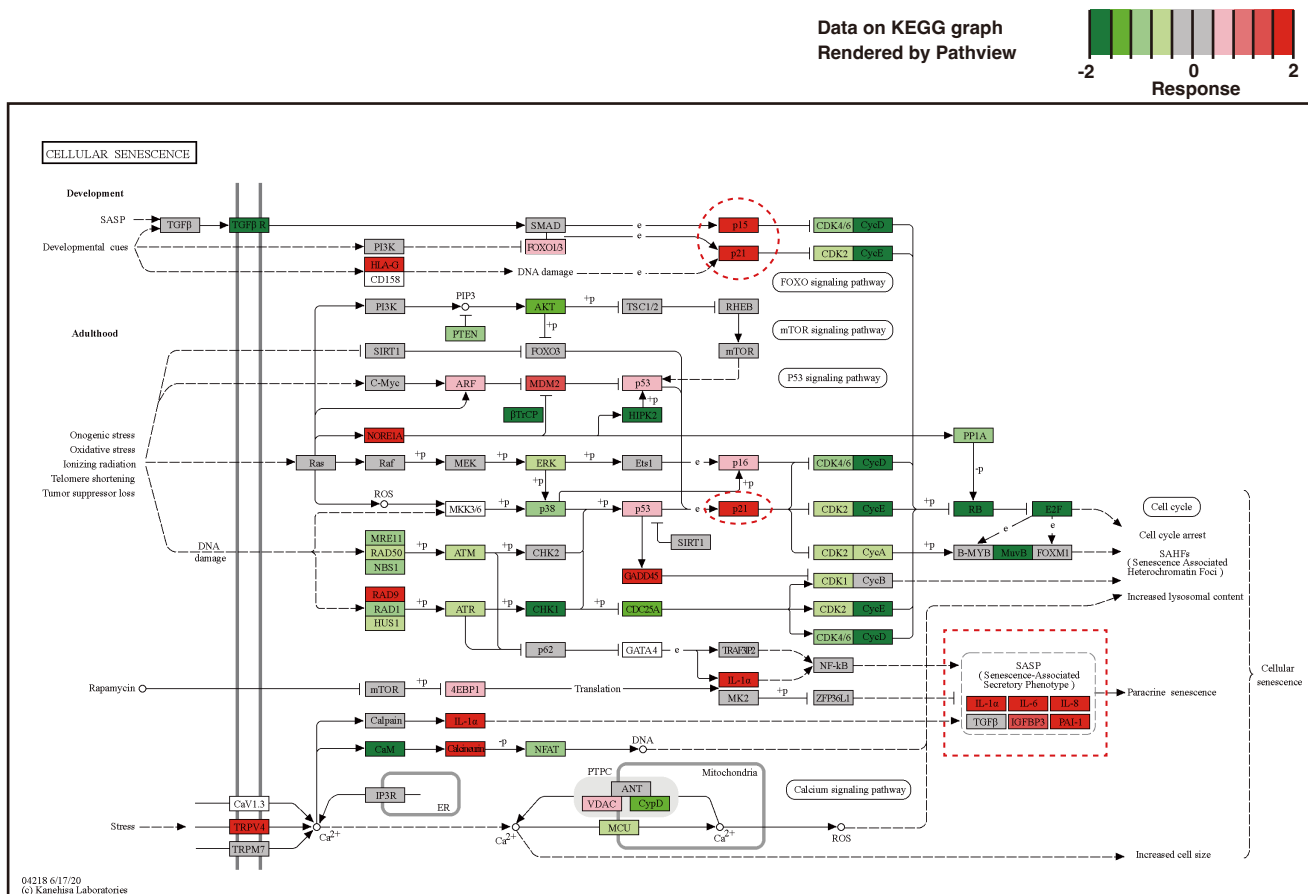

Fig. S10.

Supplement: S10 Fig — The RNA-seq data shown in S4 Fig were used. Expression levels of each gene were visualized on KEGG pathway map of “cellular senescence”, as described in the Materials and Methods. The red and green colors, according to shading, show an increase and decrease in gene expression, respectively, with D2O treatment compared to H2O treatment. Characteristic groups of genes with increased expression are surrounded by red dashed lines. (PDF) [file pone.0309689.s012.pdf]

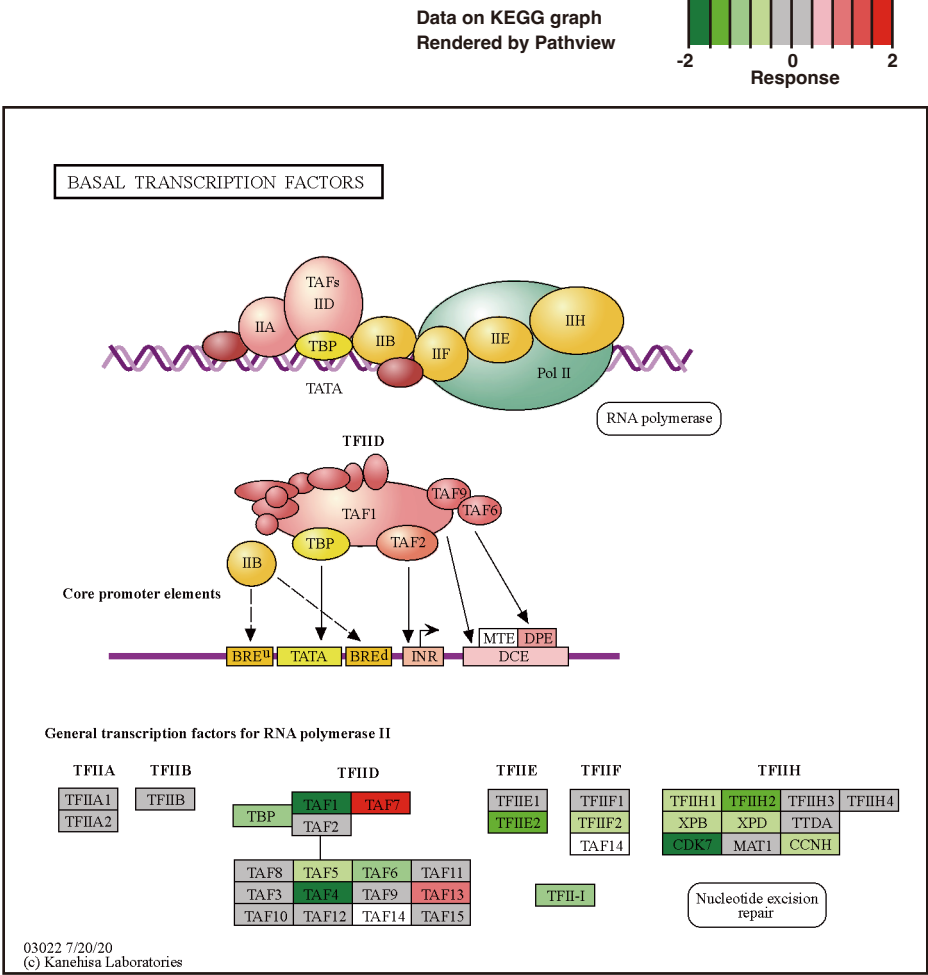

Fig. S11.

Supplement: S11 Fig — The RNA-seq data shown in S4 Fig were used. Expression levels of each gene were visualized on KEGG pathway map of “basal transcription factors”, as described in the Materials and Methods. The red and green colors, according to shading, show an increase and decrease in gene expression, respectively, with D2O treatment compared to H2O treatment. (PDF) [file pone.0309689.s013.pdf]

Data on KEGG graph  
Rendered by Pathview

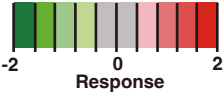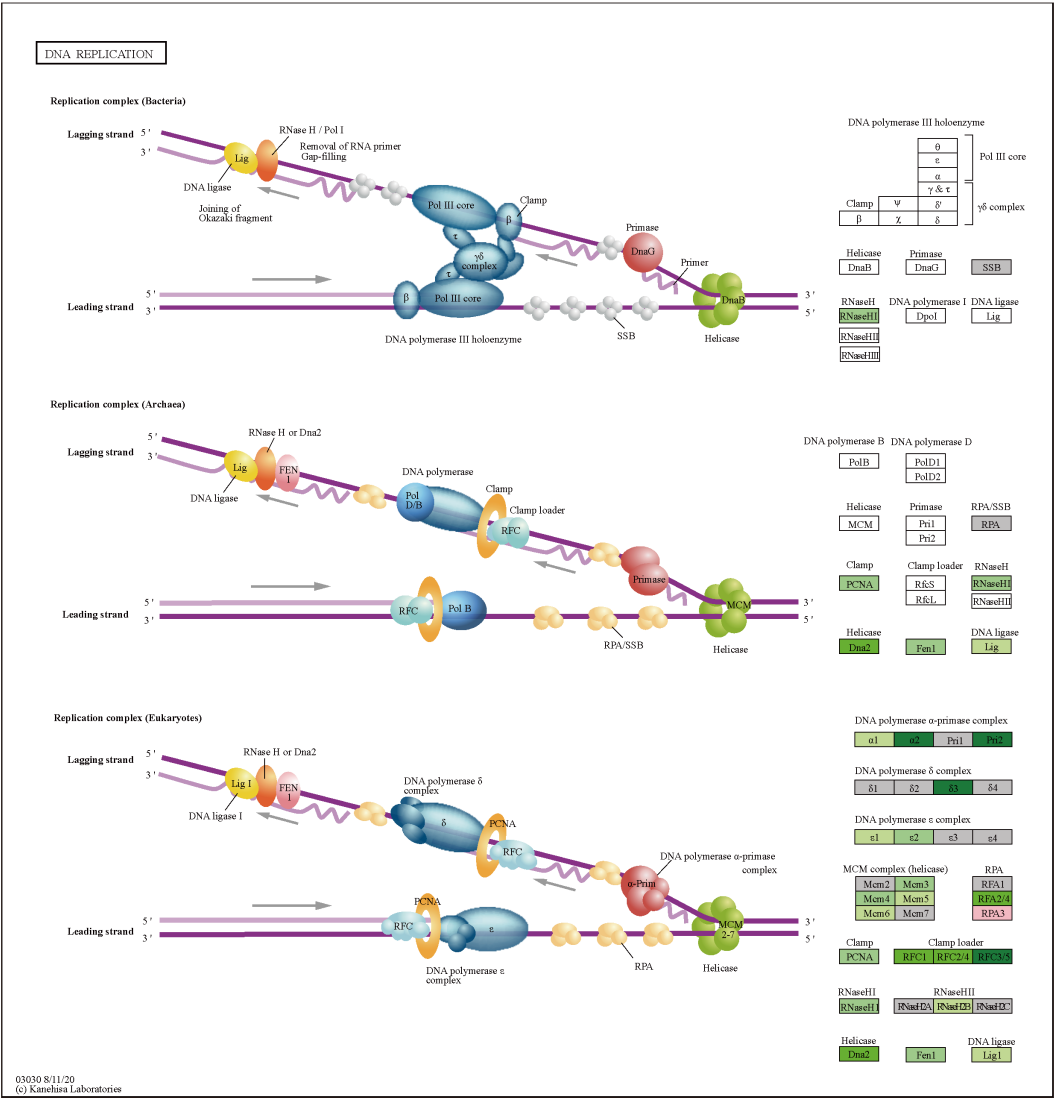

Fig. S12.

Supplement: S12 Fig — The RNA-seq data shown in S4 Fig were used. Expression levels of each gene were visualized on KEGG pathway map of “DNA replication”, as described in the Materials and Methods. The red and green colors, according to shading, show an increase and decrease in gene expression, respectively, with D2O treatment compared to H2O treatment. (PDF) [file pone.0309689.s014.pdf]

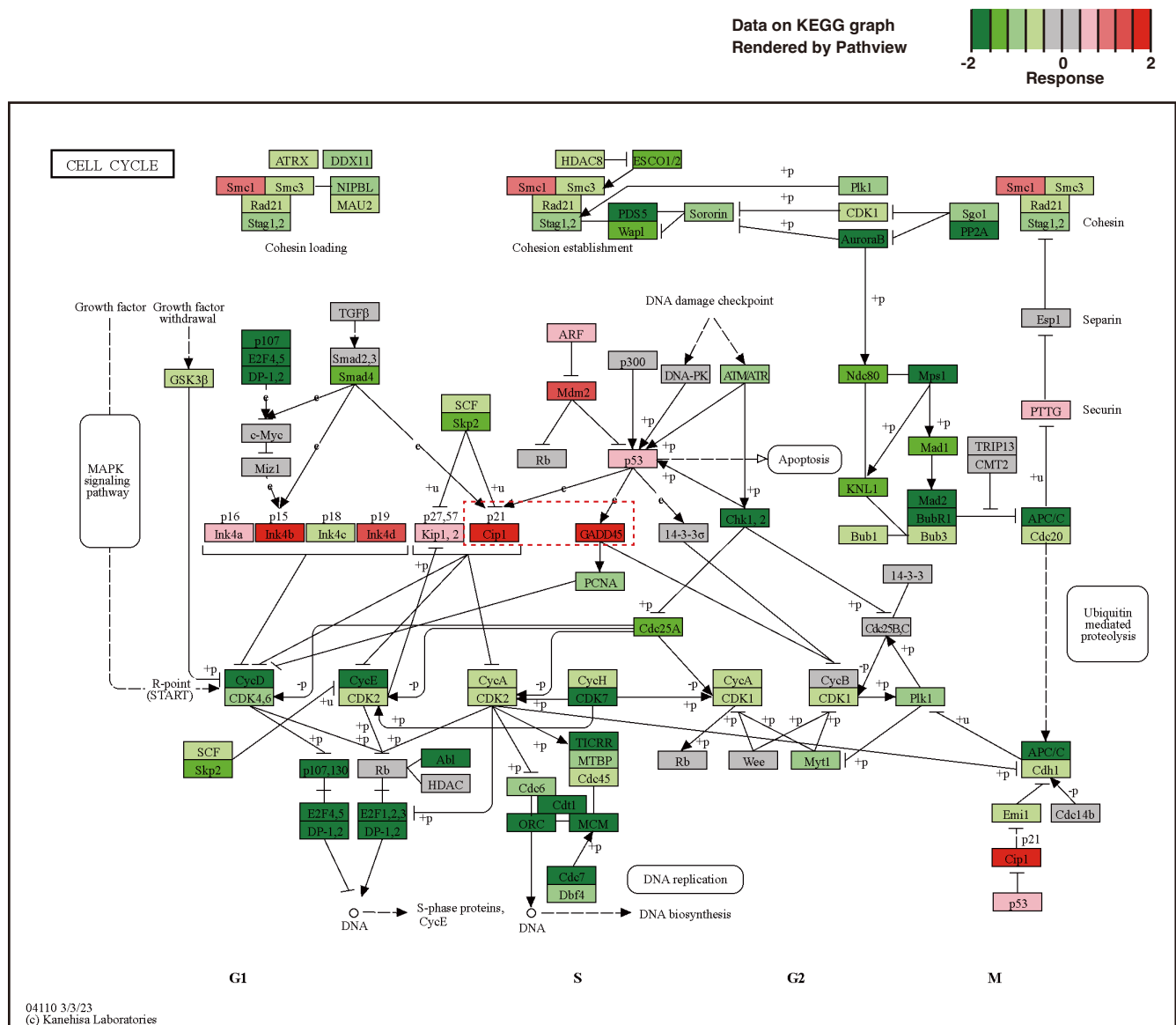

Fig. S13.

Supplement: S13 Fig — The RNA-seq data shown in S4 Fig were used. Expression levels of each gene were visualized on KEGG pathway map of “cell cycle”, as described in the Materials and Methods. The red and green colors, according to shading, show an increase and decrease in gene expression, respectively, with D2O treatment compared to H2O treatment. A characteristic group of genes with increased expression, inhibitors of cell cycle, is surrounded by red dashed lines. (PDF) [file pone.0309689.s015.pdf]

Data on KEGG graph  
Rendered by Pathview

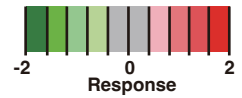

# BASE EXCISION REPAIR

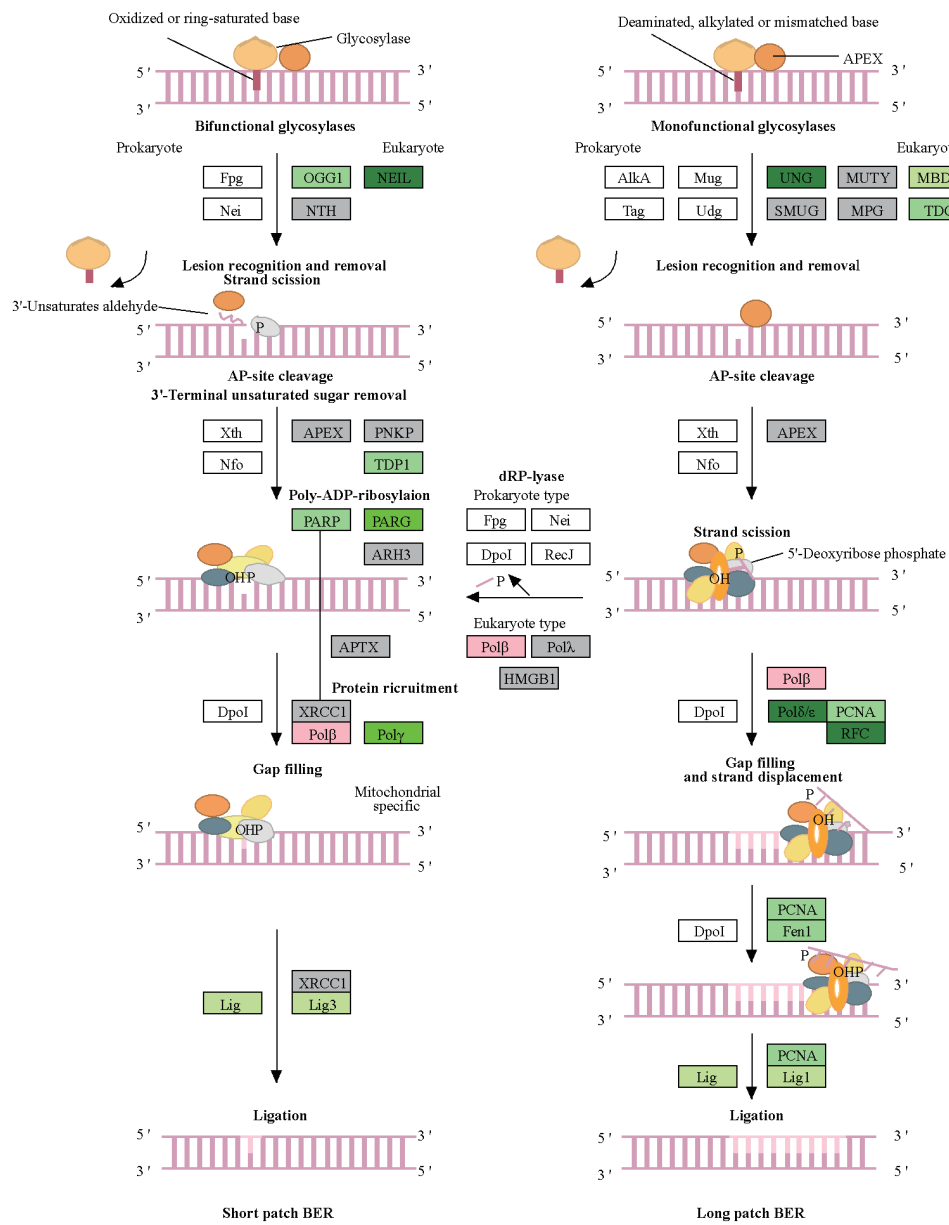

03410 3/28/23  
(c) Kanehisa Laboratories

Fig. S16.

Supplement: S16 Fig — The RNA-seq data shown in S4 Fig were used. Expression levels of each gene were visualized on a KEGG pathway map of “base excision repair”, as described in the Materials and Methods. The red and green colors, according to shading, show increased and decreased gene expression, respectively, with D2O treatment compared to H2O treatment. In the upper left part of this figure, please replace Unsaturates with Unsaturated, and then underneath that part, ribosylaion with ribosylation. Underneath that, please replace ricruitment with recruitment. (PDF) [file pone.0309689.s018.pdf]

Data on KEGG graph  
Rendered by Pathview

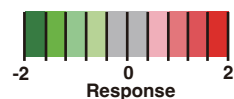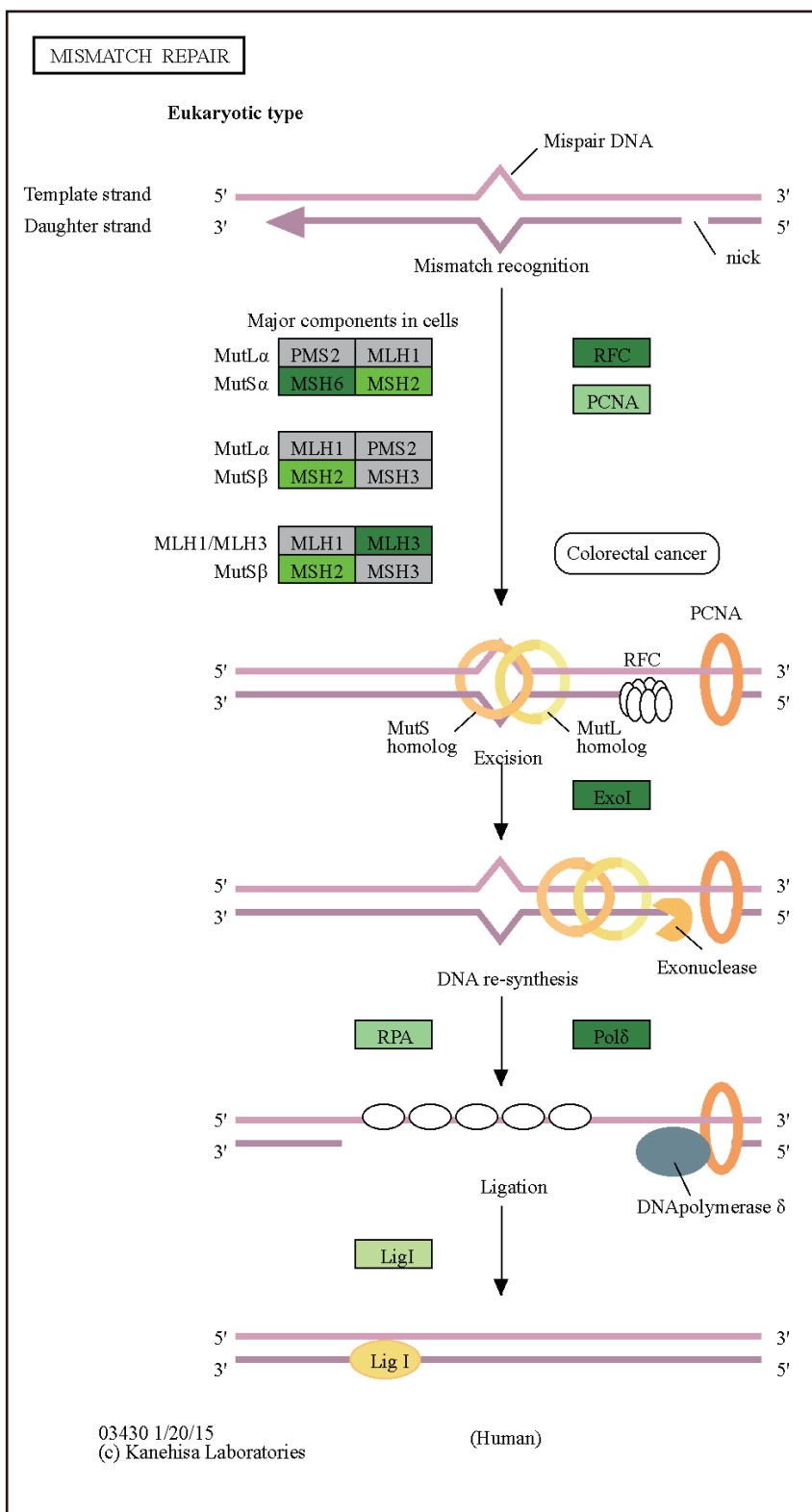

Fig. S17.

Supplement: S17 Fig — The RNA-seq data shown in S4 Fig were used. Expression levels of each gene were visualized on a KEGG pathway map of “mismatch repair”, as described in the Materials and Methods. The red and green colors, according to shading, show increased and decreased gene expression, respectively, with D2O treatment compared to H2O treatment. (PDF) [file pone.0309689.s019.pdf]

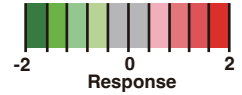

# FANCONI ANEMIA PATHWAY

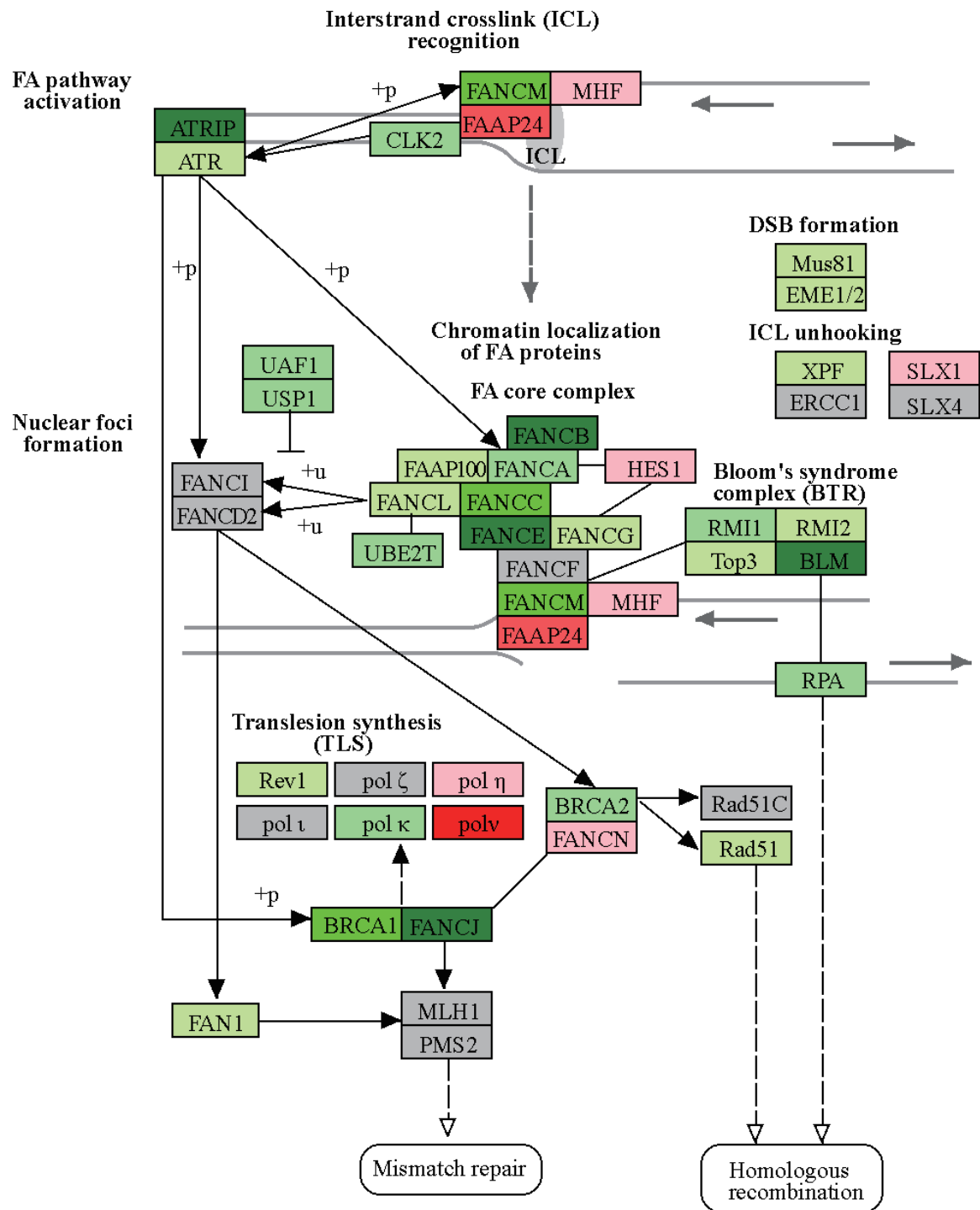

Fig. S18.

Supplement: S18 Fig — The RNA-seq data shown in S4 Fig were used. Expression levels of each gene were visualized on a KEGG pathway map of “Fanconi anemia pathway”, as described in the Materials and Methods. The red and green colors, according to shading, show increased and decreased gene expression, respectively, with D2O treatment compared to H2O treatment. (PDF) [file pone.0309689.s020.pdf]
