# Supplementary material for: Heavy water inhibits DNA double-strand break repairs and disturbs cellular transcription, presumably via quantum-level mechanisms of kinetic isotope effects on hydrolytic enzyme reactions
Source: PLoS One. 2024 Oct 3;19(10):e0309689. doi: 10.1371/journal.pone.0309689 (PMC11449287; doi:10.1371/journal.pone.0309689)
Supplement: S14 Fig — The RNA-seq data shown in S4 Fig were used. Expression levels of each gene were visualized on KEGG pathway map of “homologous recombination”, as described in the Materials and Methods. The red and green colors, according to shading, show an increase and decrease in gene expression, respectively, with D2O treatment compared to H2O treatment. A characteristic gene with increased expression, an inhibitor of HR repair, is surrounded by red dashed lines. (PDF) [file pone.0309689.s016.pdf]

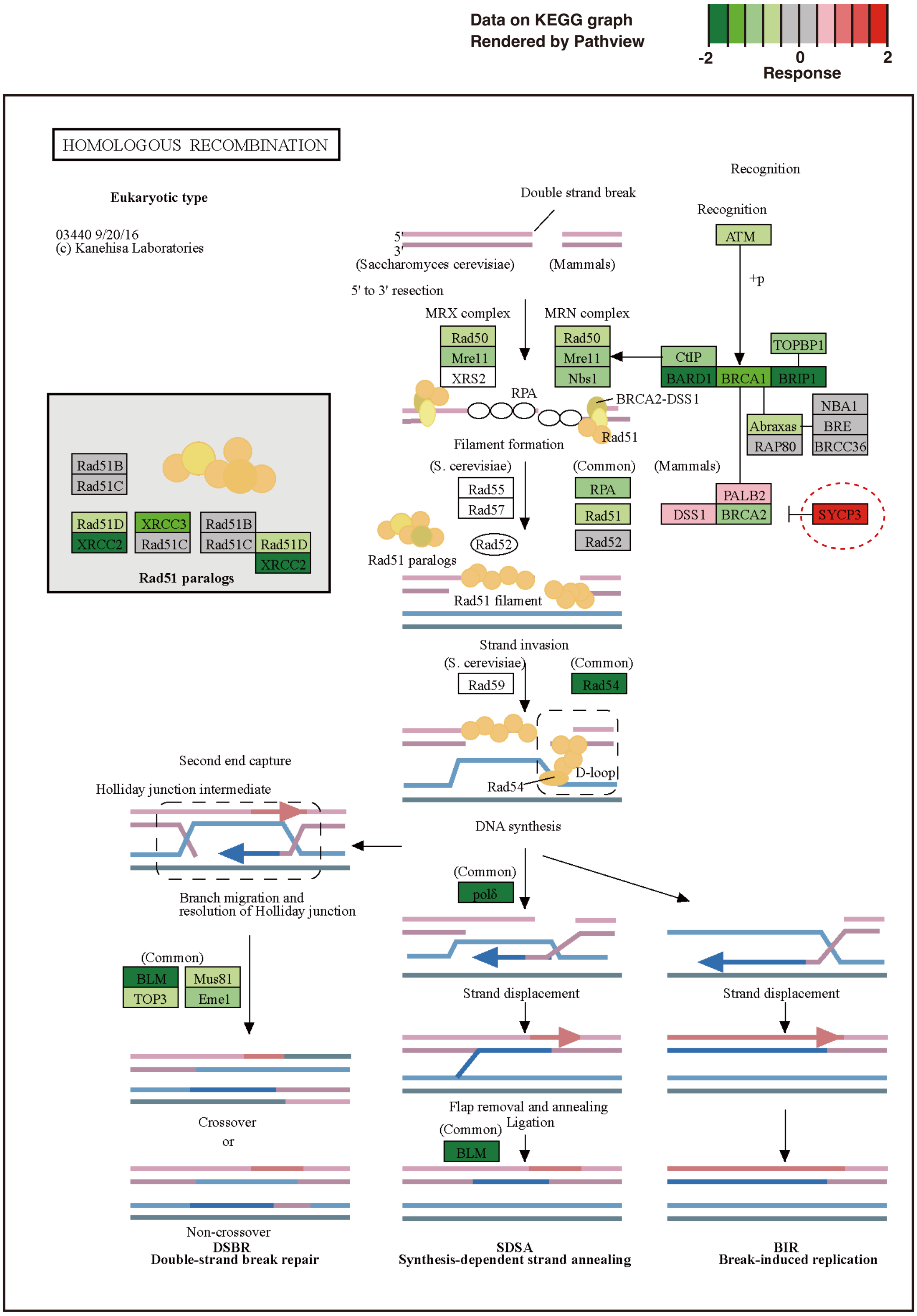
Data on KEGG graph
Rendered by Pathview
-2
0
2
Response
HOMOLOGOUS RECOMBINATION
Eukaryotic type
03440 9/20/16
(c) Kanehisa Laboratories
Double strand break
Recognition
Recognition
ATM
+p
5'
3'
(Saccharomyces cerevisiae)
(Mammals)
5' to 3' resection
MRX complex
MRN complex
Rad50
Mre11
XRS2
Rad50
Mre11
Nbs1
CtIP
TOPBP1
BARD1
BRCA1
BRIP1
RPA
BRCA2-DSS1
Rad51
NBA1
Abraxas
BRE
RAP80
BRCC36
Filament formation
(S. cerevisiae)
(Common)
(Mammals)
Rad55
Rad57
RPA
Rad51
Rad52
Rad52
PALB2
DSS1
BRCA2
SYCP3
Rad51B
Rad51C
Rad51D
XRCC2
XRCC3
Rad51C
Rad51B
Rad51C
Rad51D
XRCC2
Rad51 paralogs
Rad51 paralogs
Rad51 filament
Strand invasion
(S. cerevisiae)
(Common)
Rad59
Rad54
Second end capture
Holliday junction intermediate
Rad54
D-loop
DNA synthesis
(Common)
polδ
Branch migration and
resolution of Holliday junction
(Common)
BLM
TOP3
Mus81
Eme1
Strand displacement
Strand displacement
Flap removal and annealing
Ligation
(Common)
BLM
Crossover
or
Non-crossover
DSBR
Double-strand break repair
SDSA
Synthesis-dependent strand annealing
BIR
Break-induced replication

Fig. S14.
