# Supplementary material for: Heavy water inhibits DNA double-strand break repairs and disturbs cellular transcription, presumably via quantum-level mechanisms of kinetic isotope effects on hydrolytic enzyme reactions
Source: PLoS One. 2024 Oct 3;19(10):e0309689. doi: 10.1371/journal.pone.0309689 (PMC11449287; doi:10.1371/journal.pone.0309689)
Supplement: S15 Fig — The RNA-seq data shown in S4 Fig were used. Expression levels of each gene were visualized on a KEGG pathway map of “nucleotide excision repair”, as described in the Materials and Methods. The red and green colors, according to shading, show increased and decreased gene expression, respectively, with D2O treatment compared to H2O treatment. (PDF) [file pone.0309689.s017.pdf]

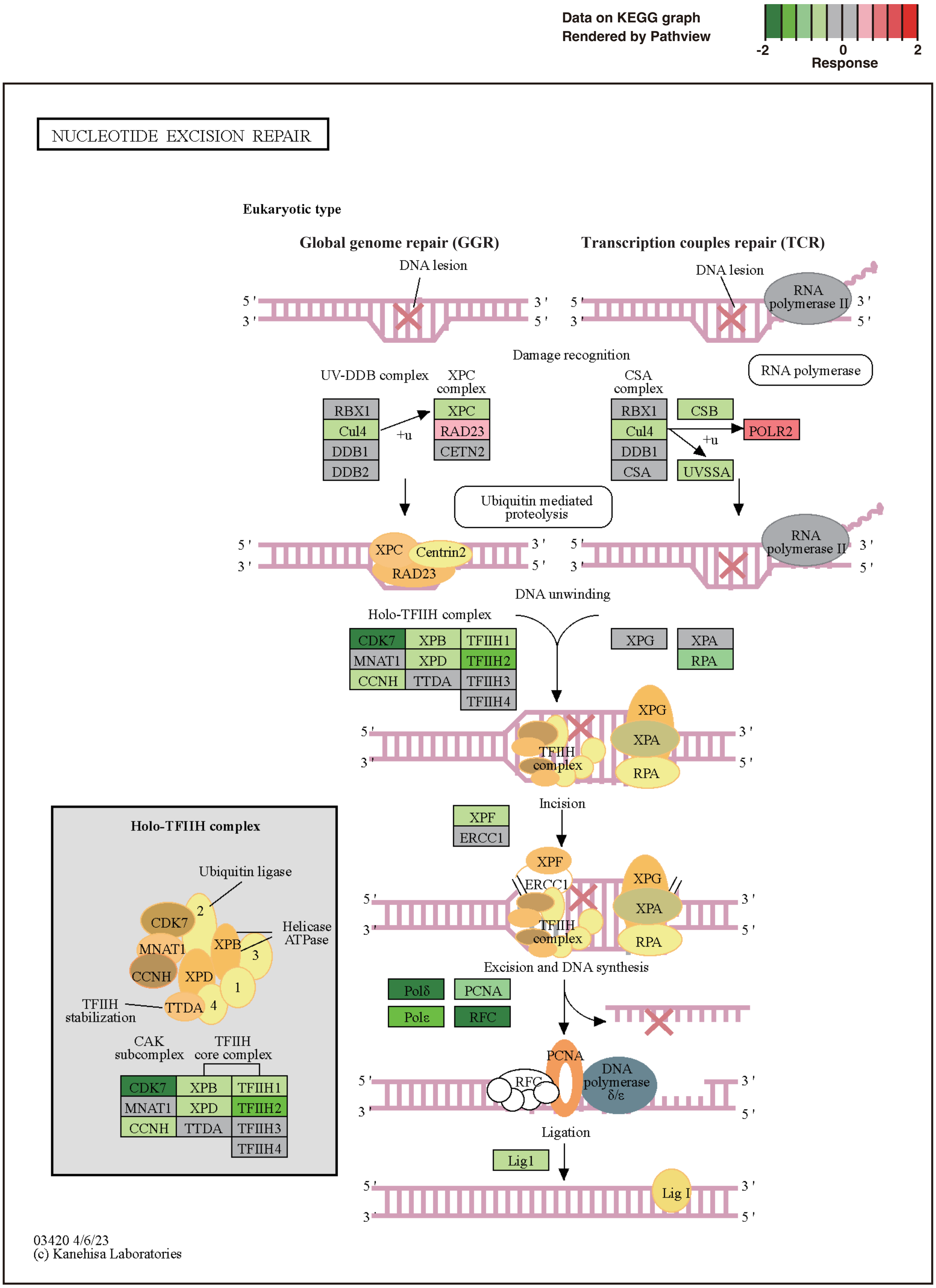
Data on KEGG graph
Rendered by Pathview
-2
0
2
Response
NUCLEOTIDE EXCISION REPAIR
Eukaryotic type
Global genome repair (GGR)
Transcription couples repair (TCR)
DNA lesion
DNA lesion
RNA polymerase II
Damage recognition
RNA polymerase
UV-DDB complex
XPC complex
CSA complex
RBX1
Cul4
DDB1
DDB2
XPC
RAD23
CETN2
+u
RBX1
Cul4
DDB1
CSA
CSB
POLR2
UVSSA
+u
Ubiquitin mediated proteolysis
XPC
Centrin2
RAD23
RNA polymerase II
DNA unwinding
Holo-TFIIH complex
CDK7
XPB
TFIIH1
MNAT1
XPD
TFIIH2
CCNH
TTDA
TFIIH3
TFIIH4
XPG
XPA
RPA
XPG
XPA
TFIIH complex
RPA
Incision
XPF
ERCC1
XPF
ERCC1
XPG
XPA
TFIIH complex
RPA
Excision and DNA synthesis
Polδ
PCNA
Polε
RFC
PCNA
RFC
DNA polymerase δ/ε
Ligation
Lig1
Lig I
Holo-TFIIH complex
Ubiquitin ligase
CDK7
MNAT1
CCNH
XPB
XPD
TTDA
Helicase ATPase
TFIIH stabilization
CAK subcomplex
TFIIH core complex
CDK7
XPB
TFIIH1
MNAT1
XPD
TFIIH2
CCNH
TTDA
TFIIH3
TFIIH4
03420 4/6/23
(c) Kanehisa Laboratories

Fig. S15.
