# Supplementary material for: Heavy water inhibits DNA double-strand break repairs and disturbs cellular transcription, presumably via quantum-level mechanisms of kinetic isotope effects on hydrolytic enzyme reactions
Source: PLoS One. 2024 Oct 3;19(10):e0309689. doi: 10.1371/journal.pone.0309689 (PMC11449287; doi:10.1371/journal.pone.0309689)

Original images used in Fig.1D

In vitro HDAC assay (37 ˚C, 20 min)

SIRT3 (μM)	-		0.03		0.05		0.1	
D_2O		+		+		+		+
H_2O	+		+		+		+	

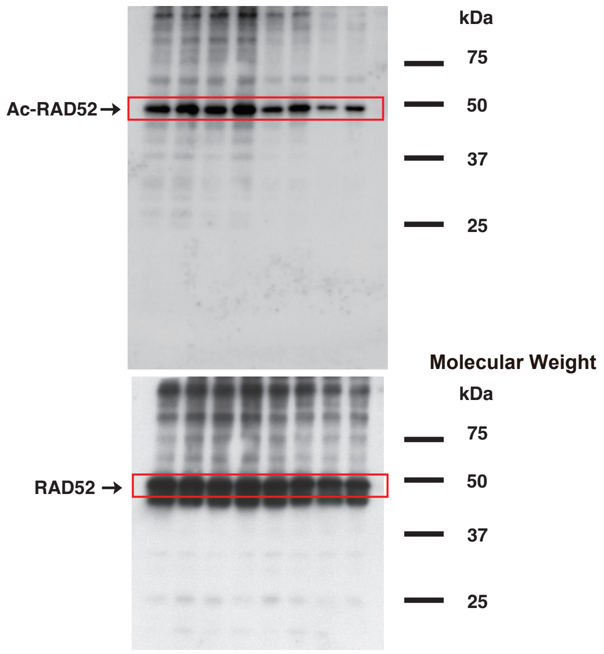

Immunoblotting

Original images used in Fig.1B

In vitro HDAC assay (30 ˚C, 40 min)

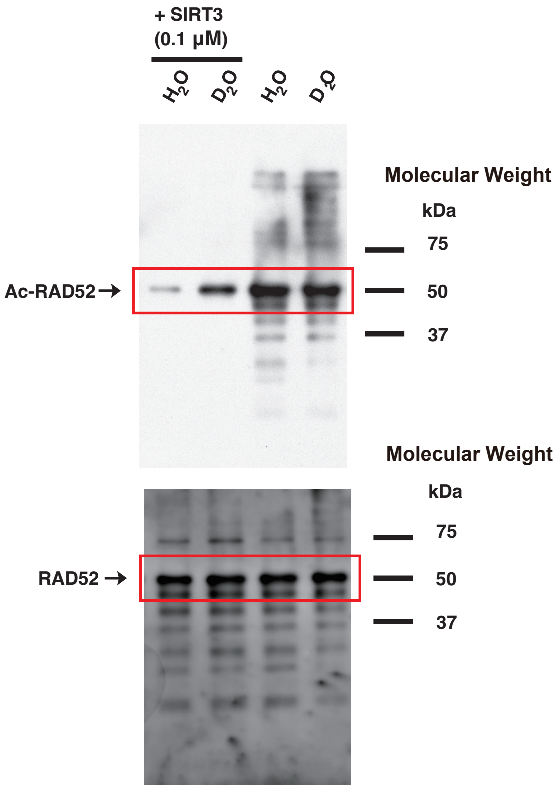

Immunoblotting

Original images used in Fig.2B

In vitro HDAC assay (15 ˚C)

Time (min)			60		90	
SIRT3 (μM)	-		0.2		0.2	
D_2O		+		+		+
H_2O	+		+		+	

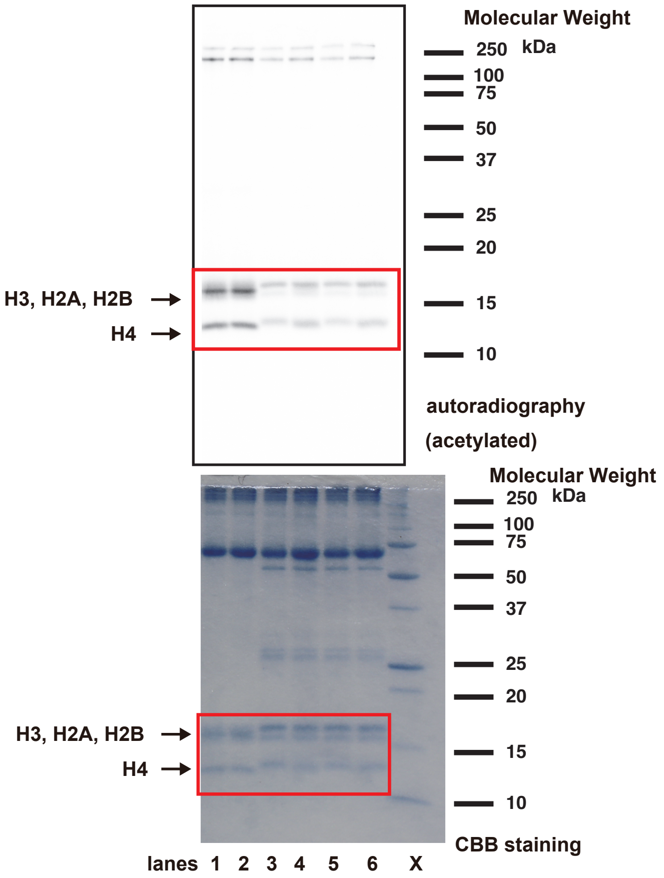

Original images used in Fig.1F

In vitro HDAC assay (37 ˚C, 40 min)

SIRT3 (μM)	-		0.1	
D_2O		+		+
H O	+		+	

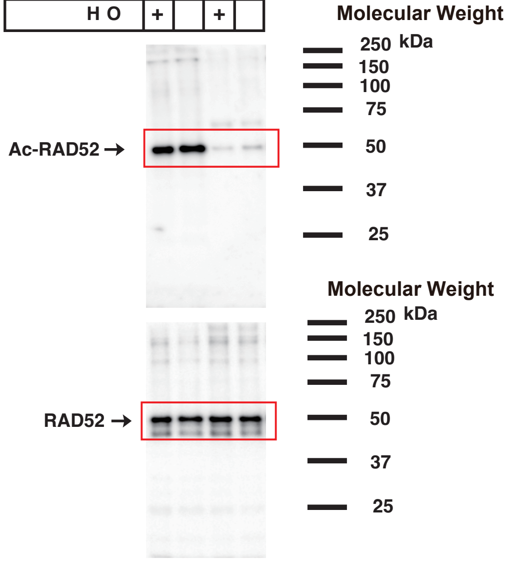

Immunoblotting

Original images used in Fig.4B

15 °C

Time (min)	0	20						60					
I-SceI (units/μl)	-	0.02		0.1		0.5		0.02		0.1		0.5	
H_2O		+		+		+		+		+		+	
D_2O			+		+		+		+		+		+

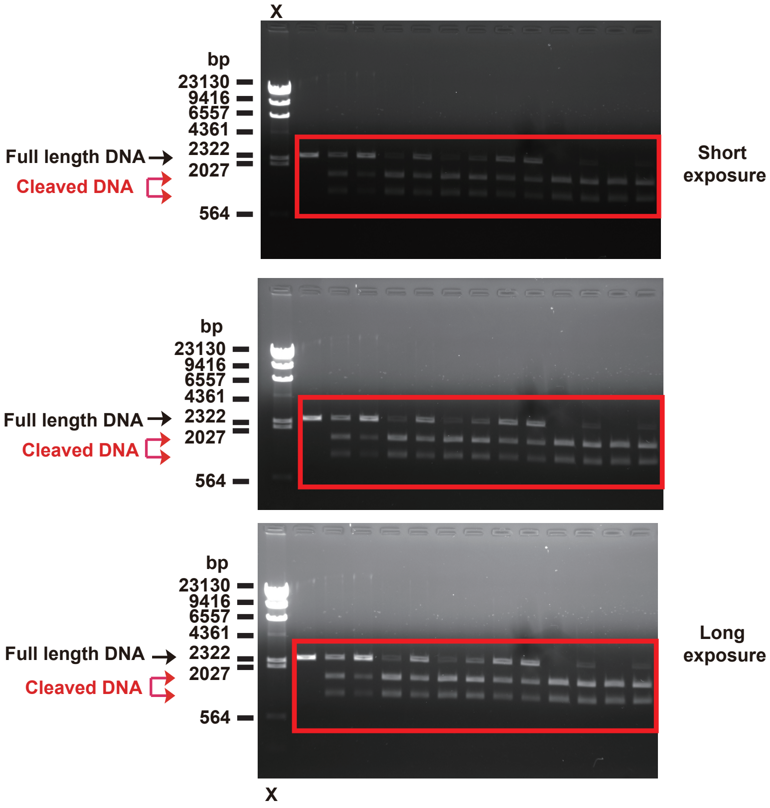

Original images used in Fig.11B

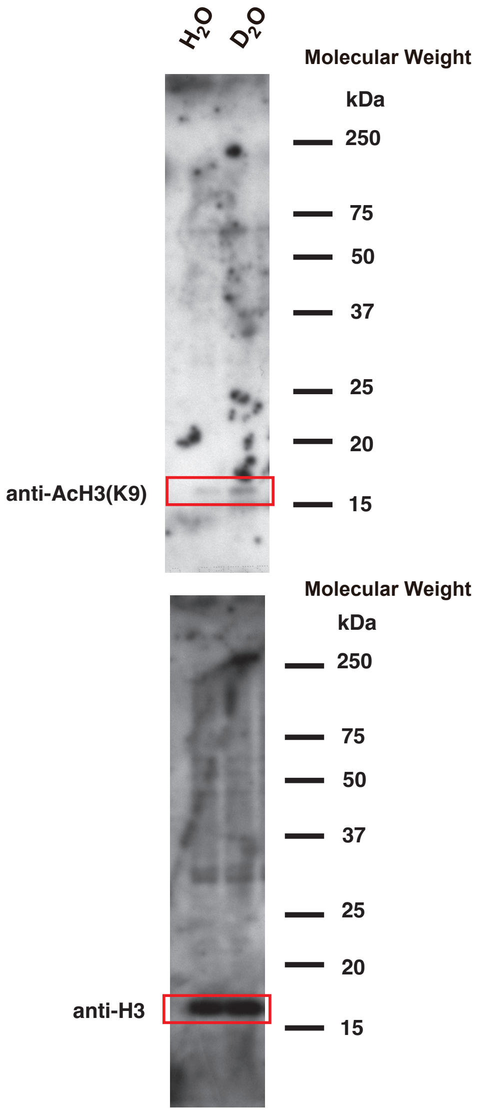

Original images used in Fig.4C

37 °C

Time (min)	0	20				60			
I-SceI (units/μl)	-	0.004		0.02		0.004		0.02	
H_2O		+		+		+		+	
D_2O			+		+		+		+

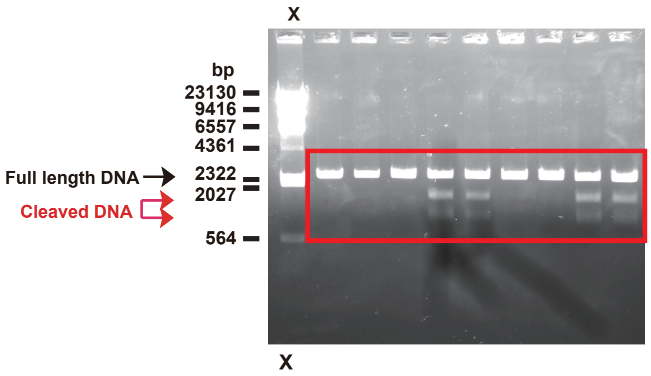

Original images used in Fig.4D

37 °C

Time (min)	0	20				60			
I-SceI (units/μl)	-	0.1		0.5		0.1		0.5	
H_2O		+		+		+		+	
D_2O			+		+		+		+

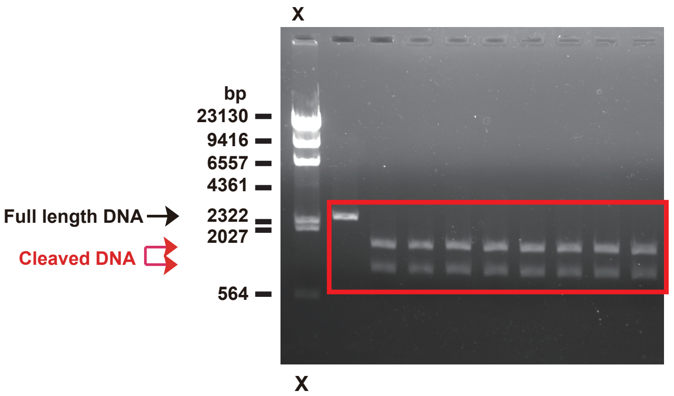

Original images used in Fig.S2A

In vitro HDAC assay

SIRT3	-					+ (0.02 μM)					+ (0.1 μM)					
D_2O				#1	#2				#1	#2				#1	#2	
H_2O	#1	#2	#3			#1	#2	#3			#1	#2	#3			X

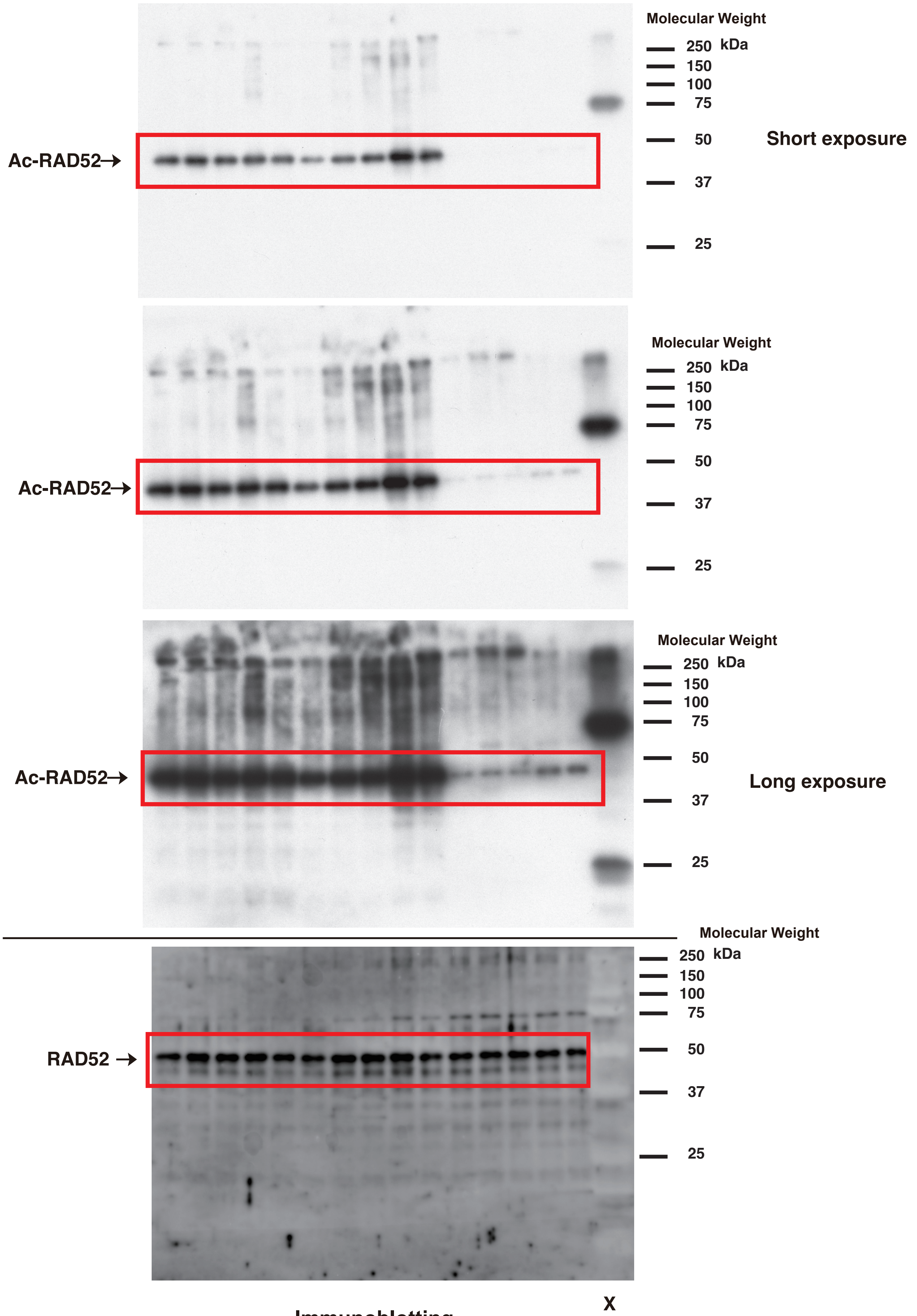

Immunoblotting

Original images used in Fig.S3A

In vitro HDAC assay (42 °C)

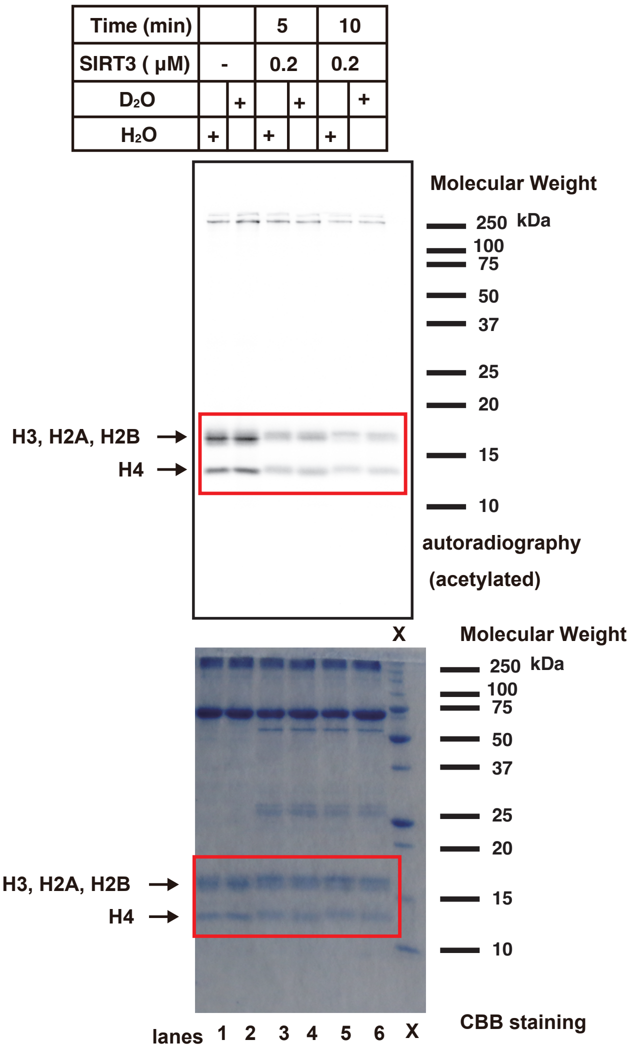

Original images used in Fig.S3D

In vitro HDAC assay (42 °C)

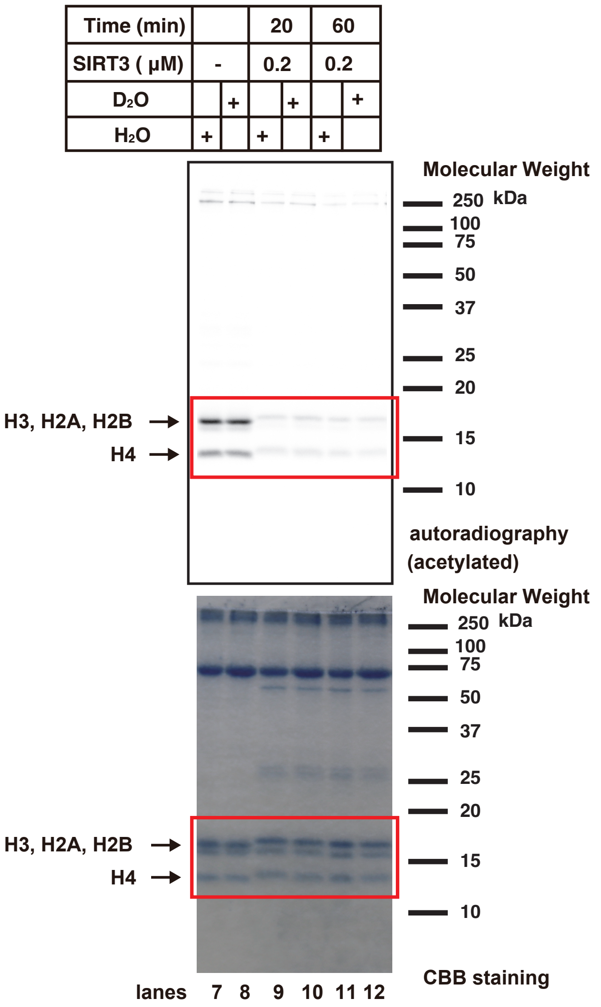

Supplement: S1 Raw image — The cropped areas used in the manuscript’s main figures and supplemental figures are surrounded by red lines. (PDF) [file pone.0309689.s023.pdf]
