# Supplementary material for: Heavy water inhibits DNA double-strand break repairs and disturbs cellular transcription, presumably via quantum-level mechanisms of kinetic isotope effects on hydrolytic enzyme reactions
Source: PLoS One. 2024 Oct 3;19(10):e0309689. doi: 10.1371/journal.pone.0309689 (PMC11449287; doi:10.1371/journal.pone.0309689)
Supplement: Attachment — Submitted filename: Heavy water inhibits DNA .....pdf [file pone.0309689.s024.pdf]

General comments to the paper entitled: Heavy water inhibits DNA double-strand break repairs and disturbs cellular transcription, presumably via quantum-level mechanisms of kinetic isotope effects on hydrolytic enzyme reactions

It is well known that the heavy hydrogen, the deuterium, is toxic in high concentrations. The author aimed to reveal the mechanism of deuterium, which is responsible for its toxic effect. To do that, the kinetic isotope effect on different enzymatic reactions was investigated in in vitro and in vivo systems. The study nicely presents the effect on several enzymatic reactions, such as deacetylation, DNA cleavage, and protein cleavage. This excellent study shows the importance of quantum mechanisms fundamentally determining the basis of life.

The critical phenomenon of quantum tunneling involves comparing the behavior of the two stable hydrogen isotopes in the chemical reaction. Due to the 100% mass difference, the quantum tunneling effect is well documented in enzymes with hydrogen transfer.

Congratulations to the authors on their excellent work relating to all the studies applying D2O in a wide range of concentrations.

However, the last sentence of the “Quantum-level effects on life phenomena” paragraph suggests the authors missed something relating to deuterium research. The “D exists as hydrogen, which is essentially negligible on the Earth…” is in line with the concept accepted for 60 years after the discovery of D; we do not need to count on living organisms.

Over 30 years ago, research started to investigate the biological effect of deuterium depletion, specifically the effect of D concentration below the natural level. Today, it is widely accepted that naturally occurring D has a central role in regulating cell growth, tumor development, and metabolism.

I strongly encourage the authors to consider adding one more paragraph to the manuscript, citing papers on deuterium-depleted water (DDW) research. The tunneling effect is present in the cells even at a natural concentration of D. The potential impact of deuterium depletion on cell growth, gene expression, and metabolism suggests the tunneling effect's importance is even higher than the present manuscript suggests. Your research has the potential to give a clear explanation of the tunneling effect for the observed findings with DDW.
